# Screening of herbal extracts binding with vascular endothelial growth factor by applying HerboChip platform

**DOI:** 10.1186/s13020-024-00987-x

**Published:** 2024-09-09

**Authors:** Yang Liu, Jia-Ming Liang, Guo-Xia Guo, Yu-Huan Qiu, Le-Le Yu, Karl Wah-Keung Tsim, Qi-Wei Qin, Gallant Kar-Lun Chan, Wei-Hui Hu

**Affiliations:** 1 College of Marine Sciences, South China Agricultural University, Guangzhou, 510642 China; 2 Nansha-South China Agricultural University Fishery Research Institute, Guangzhou, 511464 China; 3 Division of Life Science and State Key Laboratory of Molecular Neuroscience, The Hong Kong University of Science and Technology, Hong Kong, China; 4 Gallant Biotechnology Limited, Hong Kong, China; 5 Yingli (Zhongshan) Biotechnology Limited, Zhongshan, China

**Keywords:** Angiogenesis, VEGF, Chinese medicine, Herbochips

## Abstract

**Background:**

Traditional Chinese medicine (TCM) has been hailed as a rich source of medicine, but many types of herbs and their functions still need to be rapidly discovered and elucidated. HerboChip, a target-based drug screening platform, is an array of different fractions deriving from herbal extracts. This study was designed to identify effective components from TCM that interact with vascular endothelial growth factor (VEGF) as a target using HerboChip.

**Methods:**

Selected TCMs that are traditionally used as remedies for cancer prevention and wound healing were determined and extracted with 50% ethanol. Biotinylated-VEGF was hybridized with over 500 chips coated with different HPLC-separated fractions from TCM extracts and straptavidin-Cy5 was applied to identify plant extracts containing VEGF-binding fractions. Cytotoxicity of selected herbal extracts and their activities on VEGF-mediated angiogenic functions were evaluated.

**Results:**

Over 500 chips were screened within a week, and ten positive hits were identified. The interaction of the identified herbal extracts with VEGF was confirmed in cultured endothelial cells. The identified herbs promoted or inhibited VEGF-mediated cell proliferation, migration and tube formation. Results from western blotting analysis demonstrated the identified herbal extracts significantly affected VEGF-triggered phosphorylations of eNOS, Akt and Erk. Five TCMs demonstrated potentiating activities on the VEGF response and five TCMs revealed suppressive activities.

**Conclusions:**

The current results demonstrated the applicability of the HerboChip platform and systematically elucidated the activity of selected TCMs on angiogenesis and its related signal transduction mechanisms.

**Supplementary Information:**

The online version contains supplementary material available at 10.1186/s13020-024-00987-x.

## Background

Angiogenesis is the formation of new blood vessels on top of existing vessels. During angiogenesis, endothelial cells need to undergo numerous processes such as proliferation, migration, attachment, formation, remodelling and construction, but it is the vascular endothelial growth factor (VEGF) that plays a key regulatory role [1, 2]. VEGF is a family of genes including VEGF-A, VEGF-B, VEGF-C, VEGF-D, VEGF-E, and placental growth factor (PIGF), of which VEGF-A was the earliest to be discovered and the most important in angiogenesis and disease progression, Hence, VEGF-A is also referred to as VEGF [2, 3]. Studies have shown that VEGF exerts its regulatory effects by binding to the VEGF receptor (VEGFR) and activating downstream signalling pathways such as PI3K/Akt, MAPK/Erk and eNOS [4, 5]. VEGF-mediated angiogenesis is now considered essential for tumour invasion and progression [2, 5, 6]. A few studies have demonstrated that better therapeutic outcomes can be achieved by adding anti-VEGF therapeutic measures to the original treatment strategy in diseases that require constant generation of neovascularisation to achieve invasion of the surrounding tissues for progression, such as colorectal cancer, non-small-cell carcinoma and retinal macular oedema [7–9]. Therefore, inhibition of VEGF to delay disease progression is considered as a good and highly promising new strategy in the treatment strategy of the above diseases [10, 11]. Interestingly, although this therapeutic strategy in combination with anti-VEGF can achieve a synergistic effect in the clinical treatment of many diseases, anti-VEGF drugs, e.g. aflibercept, ranibizumab, sunitinib, etc., are still associated with side effects, expensive healthcare costs, and drug resistance [12, 13].

Traditional Chinese medicine (TCM), as a kind of natural plant, exists many natural compounds that have not been explored and have anticancer effects, and is regarded as a rich source of anticancer drugs [14–16]. In addition, TCM is characterised by its effectiveness, low side-effects and affordability [17]. According to TCM Simplified Integrated Database (TCMSID), many scholars have discovered, isolated and identified 20,015 natural compounds with different functions in 499 types of TCMs, including a large number of natural compounds with anticancer effects, such as Curcumae Longae Rhizoma (*Curcuma longa* L.), Polygoni Cuspidati Rhizoma et Radix (*Polygonum cuspidatum* Sieb. et Zucc.), Ginkgo Folium (*Ginkgo biloba* L.), etc. [18–21]. However, there are still many types of natural compounds and their functions that needed been precisely explored and further elucidated [22].

Numerous in vitro studies have supported the cytotoxic activities of curcumin on cancer cells, including the breast, colon, lung, ovaries, kidney and liver. Our data are consistent with previous studies that reported curcumin exerts its anticancer effects via proliferation inhibition and apoptosis induction in various cancer cell lines [23]. Polygoni Cuspidati Rhizoma et Radix has been used for treatment of inflammation, angiogenesis and infections [24, 25]. In Korea, Japan and China, Ginkgo Folium has been used as the herbal remedy for thousands of years [26]. The extract of Ginkgo Folium is commonly utilized for the treatment of a wide range of illnesses, such as arteriosclerosis and rheumatism, and Ginkgo Folium is frequently used internationally in the cosmetics, pharmacological and nutraceutical firms, with sales of > $10 billion since 2017 [27]. Currently, there is a growing interest in natural products in various countries, and a large number of natural compounds with modulatory effects on VEGF have been identified in the extracts of many kinds of TCMs [18, 24, 28–30].

The HerboChips platform is a microarray-based reverse target screening technology, which can overcome many disadvantages of traditional screening methods, such as excessive sample requirements, long separation and identification times, and complex processes [31–34]. So far, many scholars have successfully screened TCMs that can bind to nerve growth factor (NGF), tumour necrosis factor-α (TNF-α), and cytochrome P450 3A4 (CYP450 3A4) to exert specific functions using the HerboChips platform [33–35]. Inspired by this, we try using VEGF targets in conjunction with the HerboChips platform, aiming to screen TCMs that can bind to VEGF to produce specific functions, with a view to enriching the applicability of the platform and providing some theoretical references for the development of drugs for the treatment of aberrant angiogenesis or related diseases.

## Materials and methods

### Cell culture and reagents

Chemical reference substances of quercetin, kaempferol, ginkgetin, dehydrocostus lactone, panaxynol, chlorogenic acid, scutellarin, meranzin, curcumin, polydatin, resveratrol, tenuifolin and cinnamaldehyde were provided by Testing Laboratory for Chinese Medicine of HKUST. The purity of these compounds was over 98%, as detected by HPLC–DAD. HUVECs were purchased from Lonza (Basel, Switzerland) and cultured in EGM-2® BulletKit medium according to the manufacturer’s instructions (Lonza). Cell assays with HUVECs were performed using cells passaged in the laboratory to generations 3–6. Recombinant human VEGF (VEGF165) was purchased from R&D Systems (Minneapolis, MN, USA). Antibodies for Western blot were purchased from Cell Signaling Technology (Danvers, MA) and included phospho-eNOS (Ser1177), eNOS, phospho-Akt (Ser473), Akt, phospho-p44/42 MAPK (Erk1/2) (Thr202/Tyr204) and p44/42 MAPK (Erk1/2).

### Preparation of TCMs and TCMs extracts

For primary screenings, about thirty-eight kinds of TCMs, including Ginkgo Folium, Aucklandiae Radix (*Aucklandia lappa* Decne), Glehniae Radix (*Glehnia littoralis* Fr.Schmidt ex Miq.), Erigerontis Herba (*Erigeron breviscapus* (Vant.) Hand. Mazz), Murrayae Folium et Cacumen (*Murraya puniculata* (L.) Jack), Curcumae Longae Rhizoma, Polygoni Cuspidati Rhizoma et Radix, Polygalae Radix (*Polygala sibirica* L.), Myrrha (*Commiphora molmol* Engl.) and Cinnamomi Cortex (*Cinnamomum cassia* Presl), reported to regulate angiogenesis or affect related diseases were selected. These plant materials of herbs were provided by Yunnan Baiyao Group Tianzhihong Pharmaceutical Co. Ltd. (Kunming, Yunnan, China) and identified by Yunnan Institute of Materia Medica (Kunming, Yunnan, China) according to The Pharmacopoeia Commission of PR China 2020 [36]. The Voucher species were stored in Yunnan Baiyao Group Tianzhihong Pharmaceutical Co. Ltd. The TCMs extracts screened with HerboChips were prepared as follows. Firstly, 50 g of dried plant materials were ground into powder and then extracted by ultrasonic extraction with 1000 mL of 50% ethanol (1:20 w/v) for 3 days to obtain the TCMs extract. The TCMs extract was then concentrated to 2.5% of the original volume by vacuum evaporation and freeze-drying. The dried powder was stored at 4 °C until use [33].

### Preparation of the HerboChips and screening for VEGF binding activity

Fractionation of the TCMs extract by HPLC: the above TCMs extracts, including Ginkgo Folium, Aucklandiae Radix, Glehniae Radix, Erigerontis Herba, Murrayae Folium et Cacumen, Curcumae Longae Rhizoma, Polygoni Cuspidati Rhizoma et Radix, Polygalae Radix, Myrrha and Cinnamomi Cortex, were dissolved in 50% ethanol at a concentration of 50 mg/mL dissolved in 50% ethanol. The samples were separated into 96 fractions using an HPLC system equipped with a C18 column, an Agilent1100 instrument, a quaternary pump, a manual injector, a UV detector, and a workstation for identifying and measuring peaks (Agilent Technologies, USA). The wavelength was set individually using a Hypersil ODS C-18 analytical column (4.6 × 250 mm, 5 μm). The sample volume for each fractionation is 50 μL, and Constant-flow elution was performed at 30 °C using water- ACN or methanol gradient. A class of compounds were determined in each kind of herbal extract. For each microplate to represent the fraction or sample volume of 1 complete HPLC run, 150 μL of the fraction was collected at 1 min and added to the 96-well microplate. The plates are then vacuum dried using a Speed Vac (Savant, Thermo Fisher Scientific, Germany) and stored at 4 °C until use.

Preparation of the HerboChips: TCM extracts are prepared by arraying HPLC on plastic carrier chips. The HerboChips blank chip was manufactured by mode-feeding and surface activation [37]. It was briefly making two caves of plastic slides using polystyrene as a material. The surface was then successively activated with glutaraldehyde (0.4%, pH 5.0; room temperature, 4 h; washed in H_2_O), NH_4_OH (3 M, pH 11.0; 60 °C, 4 h), and 1,4-butanediol diglycidyl ether (100 mM, pH 11.0; 37 °C, overnight). Finally, the treated slides were washed with 0.1 M NaHCO_3_ (pH 8.0), sealed, dried, and stored at 4 °C until use (blank chips). Before dispensing, 30 μL of OptiFix I (40 mM sodium borate, 0.1 M PBS, 0.01% Tween 20, 20% DMSO, pH 10.0) was added to each well of the 96-well plate. Then, the samples were dispensed onto a blank chip using an automated arrayer (Biodot A101, Shuai Ran Precision, Taiwan). The control group was sequentially diluted to 4, 10, 50, and 250 ng/mL with OptiFix I. 1 μg/mL cy5-labeled streptavidin (SA-Cy5, GE Health) in OptiFix II (50 mM sodium tetraborate, 0.1 M PBS, 0.01% Tween 20, pH 7.4) was also processed with the above samples. The spotted slides were air-dried and stored at 4 degrees Celsius overnight. After blocking with blocking buffer (0.1 M ethanolamine, 0.1 M sodium tetraborate) for 1 h, the slides were rinsed four times each with TBST (50 mM Tris·HCl, 0.15 M NaCl, 0.05% Tween 20, pH 7.5) and double distilled water, air-dried for 30 min and stored at 4 °C until use. The slides that passed through the HPLC array were called HerboChips and named after the TCMs used. For example, CLR-HerboChips are a type of HerboChips arrayed with Curcumae Longae Rhizom.

Screening of VEGF binding activity in TCMs extracts by the Herbochip platform: preparation of biotinylated VEGF by dialysis method [24]. In brief, approximately 0.5 mg of VEGF (R&D systems) was dissolved in 250 μL of 100 mM NaHCO_3_, and then 23.6 μL of 10 mg/mL NHS-PEG4 biotin solution was added to the protein solution. Incubate the mixture for 2 h at 4 °C with gentle rotation. The mixture of protein solution, glycine solution, and NHS-PEG4 biotin solution were then transferred to a small glass vial, and the vial was tightly bound to the dialysis membrane with a sealing membrane around the mouth of the vial. Transfer the vial into a beaker containing phosphate-buffered saline (PBS). The whole reaction was taken at 4 °C for 48 h. At the end of the reaction, the solution sealed by the sealing film is biotinylated VEGF. Store at − 80 °C before use. TCM extracts were screened during hybridization and screening using a HerboChips-based biotinylated VEGF probe. After adding 20 ng of biotinylated VEGF per hole, the incubation was carried out at 4 °C for 1 h. Then, unbound biotinylated VEGF was rinsed with Tris-buffered solution containing 0.1% Tween 20 (TBST solution) and repeated 4×. Finally, the binding of biotinylated VEGF to the fractions was assayed by hybridization with streptavidin-Cy5™ (Invitrogen Life Technologies, Carlsbad, CA) for 1 h at 4 °C. After hybridization, streptavidin-Cy5™ was passed at 535 nm through a fluorophore microarray scanner (GenePix 4100A, Molecular Devices Corp.; Sunnyvale, CA), and the fluorescence results were further analyzed by Gene-Pix Pro 7 (version. 7.1.16) microarray scanner on the software.

### Cell viability assay

HUVECs were first inoculated in 96-well plates containing 0.1 mL of medium (5 × 10^3^/well). The following day, 0.1 mL of VEGF at a concentration of 10 ng/mL or different kinds of herbal extracts was taken instead of medium and treated for 48 h. Then, 10 μL of MTT (3-(4,5-dimethylthiazol-2-yl)-2,5 diphenyltetrazoliumbromide, Sigma-Aldrich) solution with concentration set at 5 mg/mL was added to each well and incubated for 4 h. After aspirating the medium, 150 μL of dimethylsulfoxide (DMSO, Sigma-Aldrich) was added to each well to dissolve the formed formazan salt. Finally, the intensity of color development of the formazan solution was determined using a microplate spectrophotometer at a wavelength of 570 nm, and cell viability was expressed as % of control (as 100%).

### LDH cytotoxicity assay

LDH release was determined by Sigma-Aldrich’s cellular cytotoxicity assay kit (LDH) following its instructions [38]. HUVECs were first inoculated into a 96-well plate at a density of 5 × 10^3^ per well. Then, the cell supernatants were incubated with different kinds of herbal extracts. Finally, the absorbance was determined in a microplate spectrophotometer at a wavelength of 490 nm and further analyzed. Calculation of LDH content per well, based on cytotoxicity (%) = (experimental value − low control)/(high control − low control) × 100%.

### Endothelial cell migration assay

Endothelial cell migration assays were performed in vitro wound healing [39]. HUVECs were first inoculated into a 12-well plate (20 × 10^4^/well). Once the endothelial cells were fused, a single incision was made with application of a sterile plastic pipette tip. After removal of adherent cells from the center of the cell monolayer washed by Phosphate-buffered saline (PBS), photographs were taken with a phase contrast microscope (At_0_). Fresh medium containing VEGF (10 ng/mL) with or without different kinds of herbal extracts was added to the corresponding wells, and the drug treatment was lasted for 8 h. After drug treatment, photographs were retaken in three randomly selected areas (At_8_). The wound area between the two sides of the scratch was calculated using Tscratch software (CSE Laboratories, Switzerland) after the time interval shown. Percentage of recovery = At_0_ − At_8_/At_0_ × 100%.

### Tube formation assay

Matrigel (R&D Systems, Inc.) was firstly pre-polymerized at 37 °C for 1 h. HUVECs were suspended in serum-free medium containing VEGF (10 ng/mL) with or without different concentrations of drugs and seeded into each well with cell density set at 20 × 10^4^ per well. After incubated at 37 °C for 8 h, the reticular structure of endothelial cells was observed under phase-contrast microscopy. Cells treated by serum-free medium served as a control. The percentage of inhibition rate was expressed as a percentage of control (100%). Quantitative analysis was performed by manually counting the branching points of three random fields in each well [24, 40].

### Western blot analysis

Western blot assay was performed to determine the expressions of eNOS (s1177), Akt (S473), and p44/42 MAPK (Erk1/2) (Thr202/Tyr204) proteins. Endothelial cells were incubated in medium without serum for 1 h before drug application. After drug treatment, medium was aspirated and cultures were subsequently collected in freshly prepared low-salt lysis buffer (10% glycerol, 2% SDS, 125 mM Tris–HCl, 200 mM 2-mercaptoethanol, pH 6.8) and transferred into centrifuge tubes. Next, tubes containing cell lysates were boiled at 95 °C for three times, 5 min each, and tubes were vortexed during the interval of boiling of each time. By using electrophoresis method, the protein extracts were further separated into targeted sizes of protein using 7% or 8% acrylamide gels and the gels were transferred onto nitrocellulose membranes. With voltage set at 40 V, transfection reaction was taken at 4 °C room for 15 h. Next membranes representing different sizes of protein were obtained. Then, the membranes were blocked with 5% milk solution for 1 h at room temperature. The milk solution was freshly prepared in a Tris-buffered saline containing 0.1% Tween-20 solution (TBST). After blocking assay ended, different primary antibody solutions, targeted different kinds of proteins, were used to immerse membranes at 4 °C. On the basis of the official principles of antibody application, antibodies used were diluted at a ratio of 1:1000. After primary antibody incubation for 24 h, TBST solution was used to rinse membranes for four times, 5 min each, and then membranes were reacted with horseradish peroxidase-conjugated secondary anti-rabbit antibody which was at a 1:2000 dilution. After incubated with targeted secondary antibody for 120 min at room temperature, TBST solution was used to rinse membranes for another four times, 5 min each. The reaction bands were visualized by ECL (Invitrogen) under Chemidoc imaging systems (Bio-Rad; Hercules, CA) and further analysed using Image J software. The bands of each group at 10 min were compared with the corresponding control at 0 min as to quantify the western blot in phosphorylation.

### Statistical analysis

Protein concentrations were measured by the Bradford method with a kit from Bio-Rad (Hercules, CA). For statistical analysis, the experimental data were analyzed by one way analysis of variance (ANOVA) and a Bonferroni multiple comparisons test using SPSS 16.0 software (Chicago, IL, USA). Data were showed as the mean ± standard error of the mean (SEM). Statistical significance was set at *p* < 0.05 (*), *p* < 0.01 (**), and *p* < 0.001 (***).

## Results

### Verification of biotinylated VEGF

**Fig. 1:**
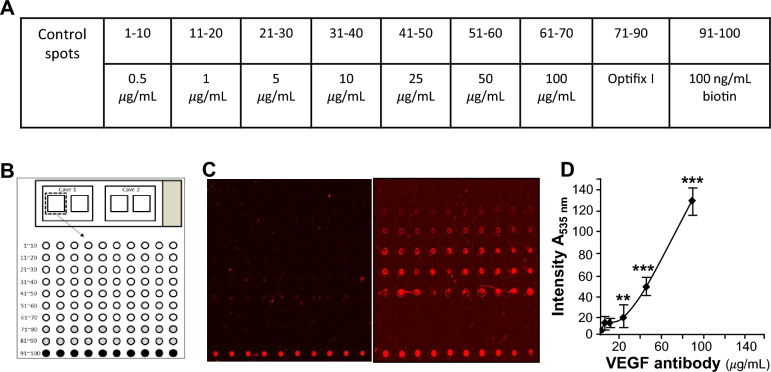
Validation of biotinylated VEGF. **A** A series concentrations of VEGF antibody were prepared. **B** VEGF antibody were dotted and fixed on a chip (HerboChips). **C** Biotinylated VEGF was hybridized with the chip. The signal was recognised by SA-Cy5 and scanned at 535 nm for fluorescence scanning. **D** Hybridization results were further quantized according to the values of fluorescence intensity. Data are expressed as X Basal, where the control was set as 1, Mean ± SEM, where* n* = 3;* p* < 0.05 (*); *p* < 0.01 (**);* p* < 0.001 (***) vs control group

To ensure that the biotinylated VEGF probe is suitable for screening TCM extracts on the HerboChip platform, we first examined the fluorescence intensity of the biotinylated VEGF probe on the VEGF antibody microarray. Biotinylated protein was used for HerboChips screening (Fig. 1). The concentration of VEGF antibody used and its formatting onto the chip were separately presented in Fig. 1A, B. After the arraying, the unreacted epoxyl groups were blocked by ethanolamine, as to reduce background staining. Different concentrations of VEGF antibody were unveiled by probing the HerboChips with biotinylated VEGF, which was then visualized by Cy5-conjugated streptavidin. Hybridization controls were provided by control spots on each chip (Fig. 1B). The samples (71–90) were used as internal negative, while samples (91–100) containing 100 ng/mL biotinamidocaproyl hydrazide were used to indicate proper binding between biotin and Cy5-conjugated streptavidin (Fig. 1A). Cave 1 on each chip was left blank (no target protein was added) to serve as an additional control for the hybridization step (Fig. 1C, left panels).

As shown in Fig. 1C, D, the signals of chips, dotted with a series of concentrations of VEGF antibody, represented different binding signal strengths (Fig. 1C). According to fluorescence intensity, the binding signals were further quantized, and the interactions of VEGF antibody with biotinylated VEGF showed a dose-dependent manner (Fig. 1D). As shown in Fig. 1, the biotinylated VEGF was suitable for the screening of herb extracts with Herbochips platform.

### Hybridization results of Herbochips

**Fig. 2:**
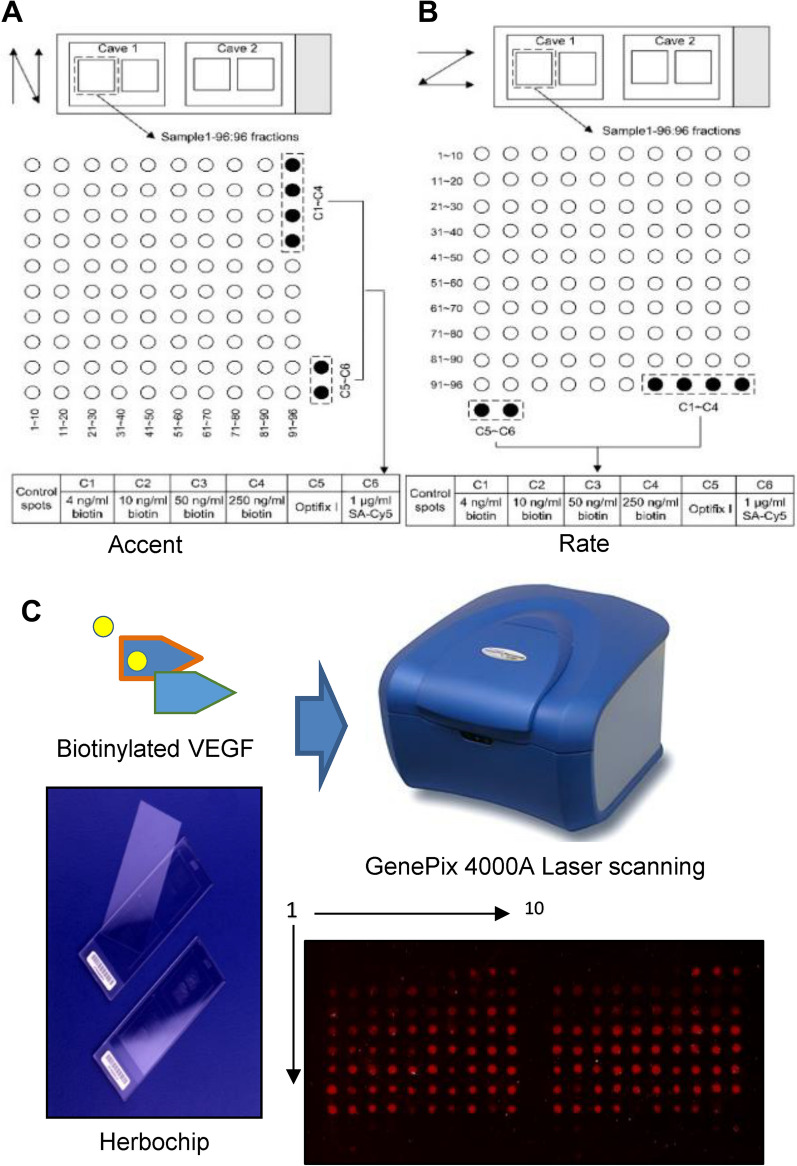
Schematic illustration of a HerboChips and screening result of Herbochips. In each of the plastic slides (25 mm × 75 mm × 1.4 mm), two caves (1 and 2) were molded and used for control and tested samples. HPLC fractions were spotted into each cave in duplicates. The 96 fractions from HPLC were formatted as spots 1–96 and there were six control spots (C1–C6) as illustrated (**A**, **B**): adopted from Huang et al. [33], showing the design of HerboChip, the quantifier and qualifier controls were included. **C** Fractions from extracts of 10 herbs were obtained by HPLC and then dotted on a chip, followed by fixation. The chip was used to hybridize with biotinylated VEGF. After hybridization with biotinylated protein target, the chip was hybridized with straptavidin-Cy5 and then went through fluorescence detection under a fluorophore microarray scanner. The left was the representative design of Herbochips including qualifier and quantifier controls. The right part was the scanning result of Herbochips using herbal extracts and hybridized with biotinylated VEGF. Representative scanned pictures under a fluorophore microarray scanner were shown

A refined Herbochips screening platform was constructed as shown in Fig. 2A, B. Two formattings of HPLC fractions onto the chip was presented. Most biological molecules including nucleic acids, proteins, and small molecules containing aforementioned functional groups would be conjugated onto the surface-activated slides (blank chips) when presented. Hybridization controls were provided by control spots on each chip (Fig. 2A, B, C1–C6). The C1–C4 samples containing different concentrations of biotinamidocaproyl hydrazide were used to indicate proper binding between biotin and Cy5-conjugated streptavidin. While C5 and C6 samples were used as internal negative and positive controls, respectively (Fig. 2A, B).

**Fig. 3:**
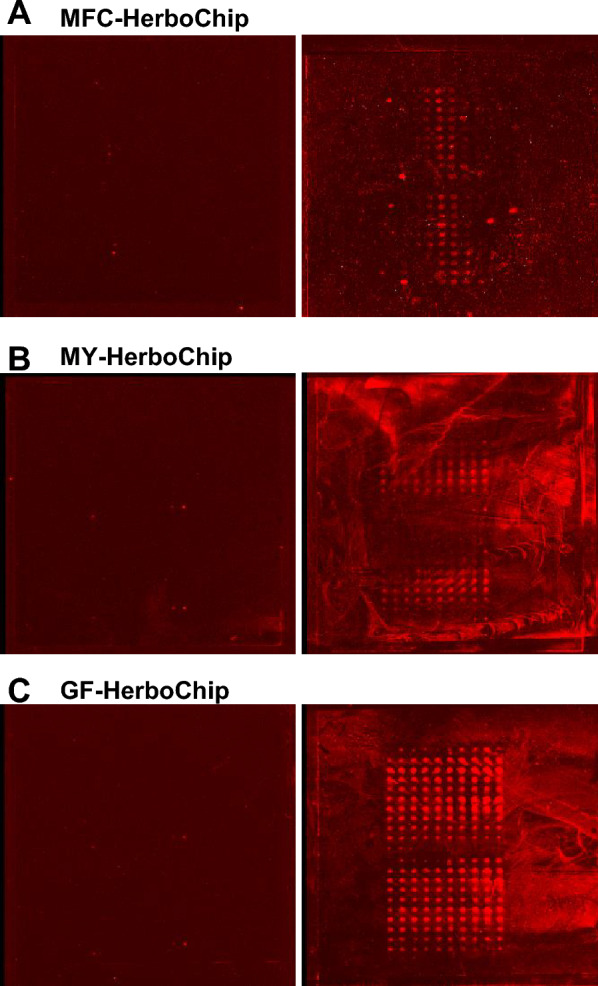
Binding signals of representative HerboChips probed by VEGF. The images were visualized by Cy5-labeled streptavidin after the binding of VEGF to **A** MFC-, **B** MY- and **C** GF-HerboChips, which were fabricated with extracts from Murrayae Folium et Cacumen, Myrrha and Ginkgo Folium, respectively. These HerboChips chosen to represent different binding signal strengths from weak (+), moderate (++), and strong (+++); as marked in Table 1

About 300 herbs, reported to have effects in angiogenesis or its related diseases, were chosen for Herbochips screening here. By applying HerboChips technology, over 300 herbs were coated onto HerboChips. Each herb was tested in five replicates to ensure consistency. After the arraying, the unreacted epoxyl groups were blocked by ethanolamine in order to suppress background. As demonstrated in Fig. 2C, HerboChips were hybridized with biotinylated VEGF. The Cy5-conjugated streptavidin was then used to react with biotinylated VEGF. Based on screening results, 38 herbal extracts, showing weak or strong fluorescence, gave positive hits in our first screening. The 38 herbs were identified to bind with VEGF. Among these positive hits, based on toxicity and accessibility, 10 widely-used herbs, including Ginkgo Folium, Aucklandiae Radix, Glehniae Radix, Erigerontis Herba, Murrayae Folium et Cacumen, Curcumae Longae Rhizoma, Polygoni Cuspidati Rhizoma et Radix, Polygalae Radix, Myrrha and Cinnamomi Cortex, were selected. The selected 10 herbs showed better signals with VEGF probe, and therefore the active fractions from corresponding herbs were further identified according to the HerboChips results. As shown in Fig. 3, the signals of three HerboChips, namely Murrayae Folium et Cacumen-, Myrrha- and Ginkgo Folium-HerboChips, were chosen to represent different binding signal strengths from weak binding (+) to strong binding (+++), and the typical pictures of the left seven kinds of HerboChips were demonstrated in Supplementary Fig. 1.

The binding activities of 10 herbs were summarized in Table 1. As shown in Table 1, Ginkgo Folium and Cinnamomi Cortex demonstrated strong interaction with VEGF, and fluorescence value was over 1000. The fluorescence value of Aucklandiae Radix, Murrayae Folium et Cacumen, Polygoni Cuspidati Rhizoma et Radix and Myrrha were between 200 and 1000, and the 4 herbs represented moderate positive hits with VEGF. Glehniae Radix, Erigerontis Herba, Curcumae Longae Rhizoma and Polygalae Radix showed weak signals with VEGF probes, and fluorescence value was less than 200.

**Table 1:** Hybridization reactivity of herbal extracts using VEGF as a probe on HerboChips

Herbal extracts	Hybridization reactivity
Ginkgo Folium	+++
Aucklandiae Radix	++
Glehniae Radix	+
Erigerontis Herba	+
Murrayae Folium et Cacumen	+
Curcumae Longae Rhizoma	+
Polygoni Cuspidati Rhizoma et Radix	++
Polygalae Radix	+
Myrrha	++
Cinnamomi Cortex	+++

The type of schematic illustration for each kind of herb extract was shown, including its related hybridization reactivity. “+” represented weak positive, which corresponding fluorescence value (FV) was less than 200; “++” represented moderate positive, which the FV was between 200 and 1000; “+++” represented strong positive, which the FV was over 1000

### HPLC profile of herbal extracts

**Fig. 4:**
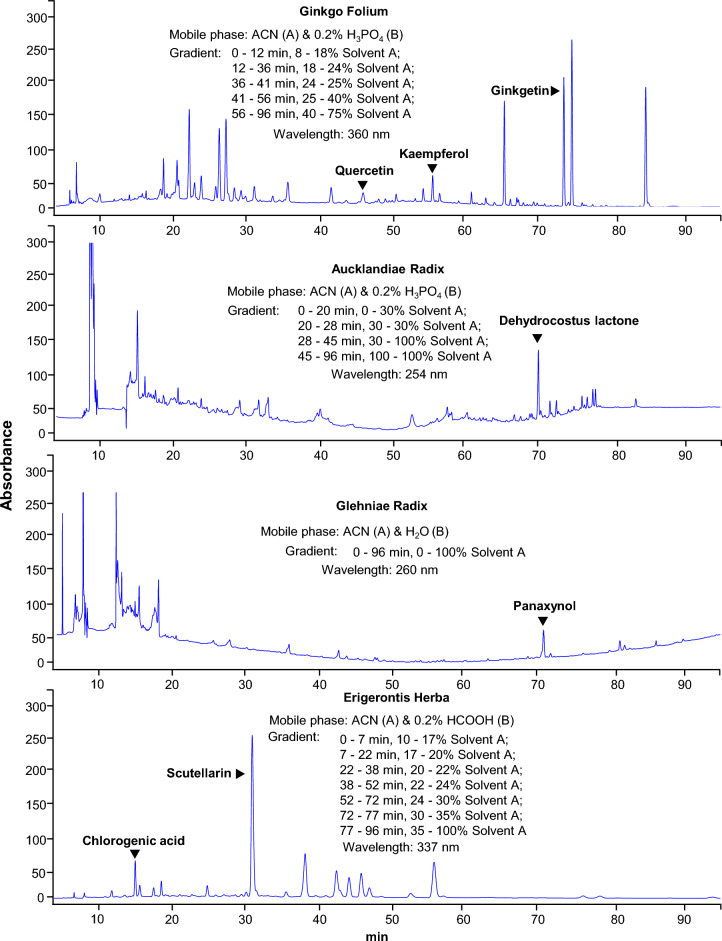

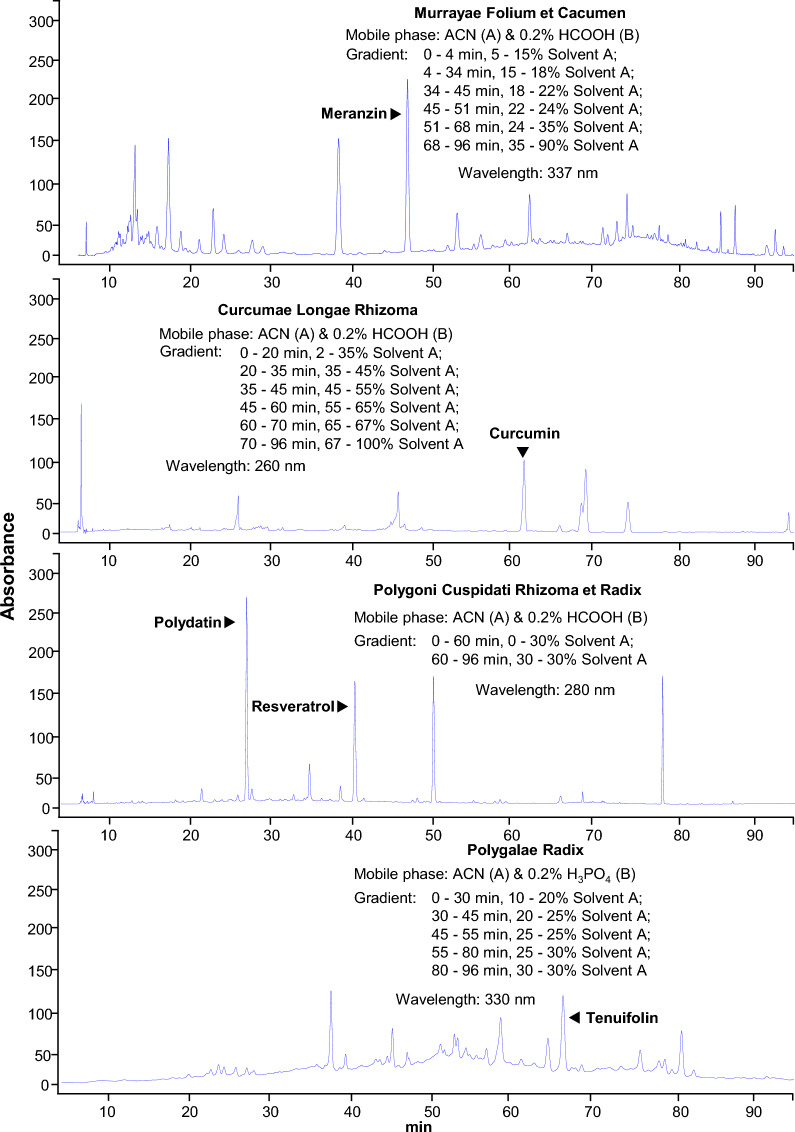

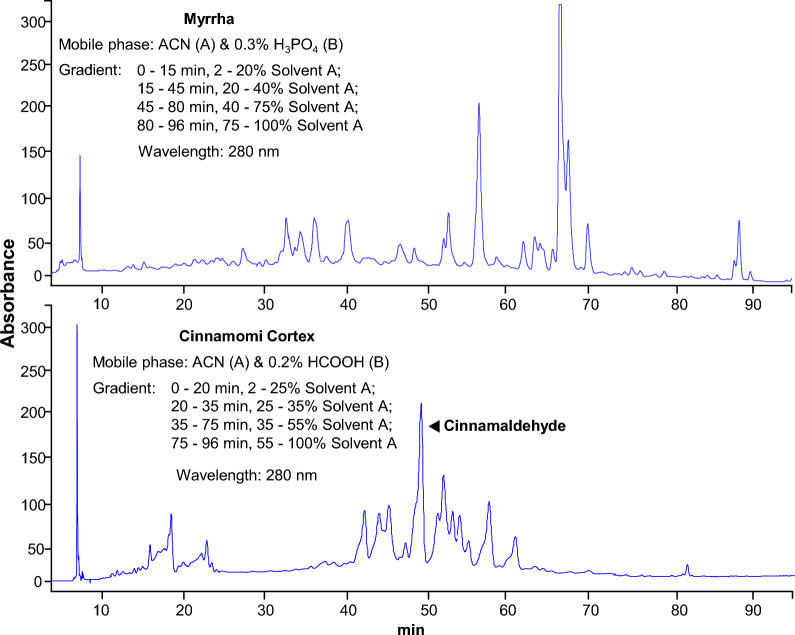
HPLC fingerprint chromatograms for ten herbs. Fifty gram dried plant materials were made into powder and extracted by sonification successively with 1000 mL of 50% ethanol (1:20 w/v) for 72 h. The vacuum evaporation was used to concentrate the freshly-obtained extract to 2.5% of its initial volume and then dried in the lyophilizer. 100 mg freeze-dried powder was weighed into a 15-mL centrifugal tube, and 4 mL of 50% methanol was added for sonication for 15 min. The supernatant was centrifuged at 1000×*g* for 5 min before analysis. Ten microlitre of the supernatant was injected into HPLC system for each kind herb. The resolution of markers for each herbal extract was over 1.5. The chromatographic patterns of the ten herbs were revealed, *n* = 3

The selected 10 herbs were extracted separately and the extraction efficiency was different. The highest extraction efficiency was 22.8% in Ginkgo Folium, while the extraction efficiency of Murrayae Folium et Cacumen was 5.3%. The extraction efficiency for other 8 herbs was between 5.3 and 22.8%. Analytical method was separately established to obtain representative fingerprint chromatogram of each kind of ethanol extract, which could be employed for comparison of different batches of herbs. The analytic column was Grace VisionHT C18 column (C18 column, 4.6 × 250 mm, 5 μm) for its broad-spectrum applicability to various types of natural compounds. The detection wavelength was set from 190 to 400 nm to detect various substances. The gradient elution programs covered 0–100% acetonitrile and were set mainly to separate water-soluble substances. A series of gradient elution were utilized separately for each kind of herbal medicine as shown in Fig. 4.

The HPLC fingerprints of ethanol extracts from the selected herbs showed that there were plenty of trace compounds. By analysing the HPLC profiles, the ethanol extracts in general contained high amounts of trace compounds, recognized by number of peaks (Fig. 4). This chemical analysis was to ensure the repeatability of all biochemical assays as described below.

### TCMs affect VEGF-mediated endothelial cell proliferation, migration and tube formation in vitro

**Fig. 5:**
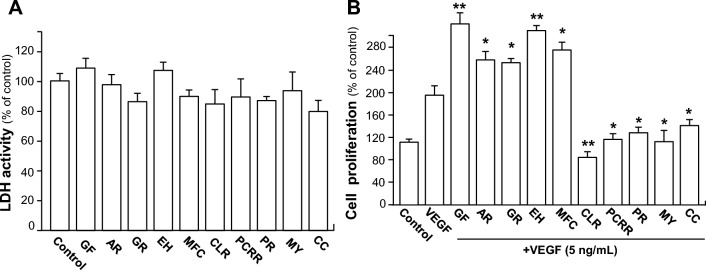
TCMs exert effects on VEGF-induced cell proliferation. **A** Endothelial cells seeded in 96-well plates, at a density of 5 × 10^4^ cells/well, were treated with ten herbs separately at different concentrations for 48 h without VEGF, the supernatants of HUVECs were collected for LDH cytotoxicity assay. **B** The treatment was similar to **A** except with VEGF (5 ng/mL). After the treatment of Ginkgo Folium (GF), Aucklandiae Radix (AR), Glehniae Radix (GR), Erigerontis Herba (EH), Murrayae Folium et Cacumen (MFC), Curcumae Longae Rhizoma (CLR), Polygoni Cuspidati Rhizoma et Radix (PCRR), Polygalae Radix (PR), Myrrha (MY) and Cinnamomi Cortex (CC), the viability of cell was determined by MTT assay. Data are expressed as Mean ± SEM of the percentage of change as compared with control, where *n* = 4; *p* < 0.05 (*); *p* < 0.01 (**); *p* < 0.001 (***) vs VEGF-treated group

The TCM extracts that gave positive hits were further analyzed in cultured endothelial cells. Cell viability tests were performed to determine the maximum possible concentration of herb extracts that could be used in functional assays (Table 2). In general, the cells showed no morphological changes at concentrations under 0.1 mg/mL. An assay for LDH release was performed. As shown in Supplementary Fig. 2 and Fig. 5A, the concentrations of 10 herbs being used here exerted no toxicity effects on endothelial cells.

**Table 2:** The summary table of TCMs on the concentration applied on VEGF-induced angiogenic functions and related signaling study

Code	Official name	Concentration (µg/mL)
GF	Ginkgo Folium	100
AR	Aucklandiae Radix	50
GR	Glehniae Radix	100
EH	Erigerontis Herba	100
MFC	Murrayae Folium et Cacumen	50
CLR	Curcumae Longae Rhizoma	100
PCRR	Polygoni Cuspidati Rhizoma et Radix	100
PR	Polygalae Radix	100
M	Myrrha	100
CC	Cinnamomi Cortex	30

The maximum possible concentration of herb extracts that could be used in functional assays

To elucidate the anti-angiogenic effects of selected herbs in vitro, the VEGF-induced endothelial cell proliferation was determined. VEGFA, simply denoted as VEGF, was being used here for analysis; because which was reported to activate HUVEC via VEGFR2 [1]. As shown in Fig. 5B, extracellular applied VEGF worked as a formidable attractant for HUVECs proliferation at ~ 60% of increase. The proliferation of HUVECs, induced by VEGF, was significantly potentiated after treatment of Ginkgo Folium, Aucklandiae Radix, Glehniae Radix, Erigerontis Herba and Murrayae Folium et Cacumen, while herbs, including Curcumae Longae Rhizoma, Polygoni Cuspidati Rhizoma et Radix, Polygalae Radix, Myrrha and Cinnamomi Cortex, showed significant suppressive effects on VEGF-induced endothelial cell proliferation (Fig. 5B). It could be concluded that the 10 herbs at non-toxic effect doses could remarkably potentiate or inhibit cell proliferation, triggered by VEGF, in cultured HUVECs.

**Fig. 6:**
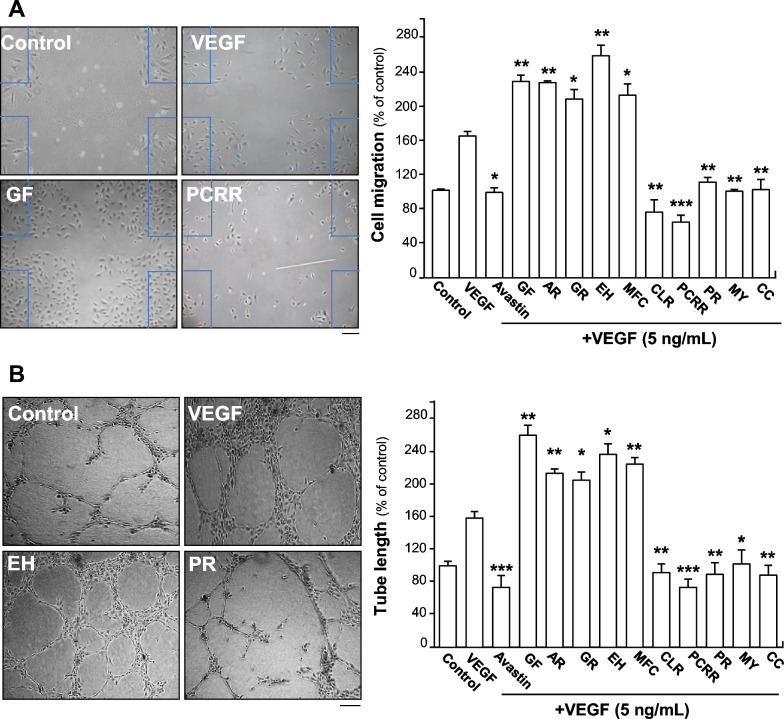
TCMs affect VEGF-mediated endothelial cell migration. **A** HUVECs, at a density of 20 × 10^4^ per well, were seeded in a 12-well plate. The artificial wounds of cells were made by scratching cell monolayer mechanically. Pictures of wounds were taken separately at 0 h and 8 h after scratching by using a phase-contrast microscope (left panel). Various treatments of 5 ng/mL VEGF, separately with or without different concentrations of GF, AR, GR, EH, MFC, CLR, PCRR, PR, MY, CC and 200 μg/mL Avastin, were performed. Avastin was used as positive control. Abbreviations of herbs were described as in Fig. 5. The blue lines roughly represented the scratch position of the cells at 0 h. **B** A total of 20 × 10^4^ HUVECs were seeded into each well of a 12-well plate which was pre-coated with matrigel. VEGF (5 ng/mL), separately with or without indicated concentrations of GF, AR, GR, EH, MFC, CLR, PCRR, PR, MY, CC and 200 μg/mL Avastin, was added together with cell suspension for additional 8 h. Images of endothelial cells network-like structures were examined under a phase-contrast microscope, and the existing branching points in three fields selected randomly per well were counted to quantify the endothelial cells tube formation (left panel). Abbreviations of herbs were described as in Fig. 5. Data are expressed as the percentage of change as compared to control in Mean ± SEM (right panel), where *n* = 3; *p* < 0.05 (*); *p* < 0.01 (**); *p* < 0.001 (***) vs VEGF-treated group. Bar = 40 μm

Endothelial cell migration and tube formation play important roles in cell growth and progression. Therefore, we studied the effects of 10 herbs on the potential capacity of endothelial cells in metastatic in vitro. Avastin (Roche, Basel, Switzerland), the trade name of Bevacizumab, is an antibiotic protein and works as a medication used to treat a number of types of cancers by its binding to VEGF. Avastin was here used as a positive control for its inhibitory effect of angiogenesis [41, 42]. Scratch movement test was performed to determine endothelial cell migration after herbal medicine treatment. Application of VEGF promoted the endothelial cell migration, as shown in Fig. 6A. However, the separate treatment of Ginkgo Folium, Aucklandiae Radix, Glehniae Radix, Erigerontis Herba, Murrayae Folium et Cacumen, at indicated concentrations, effectively potentiated VEGF-induced endothelial cell migration by ~ 40% to ~ 80%, while the separate treatment of Curcumae Longae Rhizoma, Polygoni Cuspidati Rhizoma et Radix, Polygalae Radix, Myrrha and Cinnamomi Cortex significantly inhibited the VEGF-mediated endothelial cell migration by ~ 35% to ~ 90% (Fig. 6A). The typical pictures of the Avastin-treated group and the other eight kinds of herbs-treated groups were shown in Supplementary Fig. 3.

To further study pharmaceutical effects of TCMs on angiogenesis, tube formation assay was performed to explore whether the selected 10 herbs could affect VEGF-mediated the formation of capillary-like tube in endothelial cells. After treatment with VEGF (5 ng/mL), the robust and elongated tube-like structures were obtained. After medicine treatment, more robust and elongated tube-like structures were obtained with application of different concentrations of Ginkgo Folium, Aucklandiae Radix, Glehniae Radix, Erigerontis Herba and Murrayae Folium et Cacumen. The potentiating efficiency of aforementioned 5 herbs increased by ~ 50% to ~ 100%, compared with VEGF-treated group. However, a prominent interruption in capillary-like tube was captured by exposing to different concentrations of Curcumae Longae Rhizoma, Polygoni Cuspidati Rhizoma et Radix, Polygalae Radix, Myrrha and Cinnamomi Cortex (Fig. 6B): this effect was similar to inhibition of VEGF-triggered endothelial cell migration, and the range of inhibitory rate was from ~ 40 to ~ 70%, as compared with VEGF-treated group. Avastin decreased VEGF-induced tube formation by ~ 90%, and showed similar inhibition as compared that of Curcumae Longae Rhizoma, Polygoni Cuspidati Rhizoma et Radix, Polygalae Radix, Myrrha and Cinnamomi Cortex (Fig. 6B). These results therefore indicated that the extracts of Ginkgo Folium, Aucklandiae Radix, Glehniae Radix, Erigerontis Herba and Murrayae Folium et Cacumen showed robust potentiating activity to VEGF response, while the extracts of Curcumae Longae Rhizoma, Polygoni Cuspidati Rhizoma et Radix, Polygalae Radix, Myrrha and Cinnamomi Cortex suppressed the VEGF-mediated responses. The representative pictures of the Avastin-treated group and the other eight kinds of herbs-treated groups were demonstrated in Supplementary Fig. 3.

### The effects of TCMs on VEGF-mediated signaling

To further explore the molecular mechanisms of 10 herbs in regulating VEGF-induced responses in endothelial cells, we studied VEGFR2 and its downstream signalling, e.g. Erk, PI3-K/Akt and eNOS in the presence of VEGF and herbal extracts [43]. Previous studies showed that VEGFR2 phosphorylation could subsequently cause activation of its multiple relevant downstream signals, i.e. endothelial cells proliferation and differentiation activities [44]. The multiple necessary downstream signaling elements involving in VEGFR2 activation were selected to be determined by western blotting assay.

**Fig. 7:**
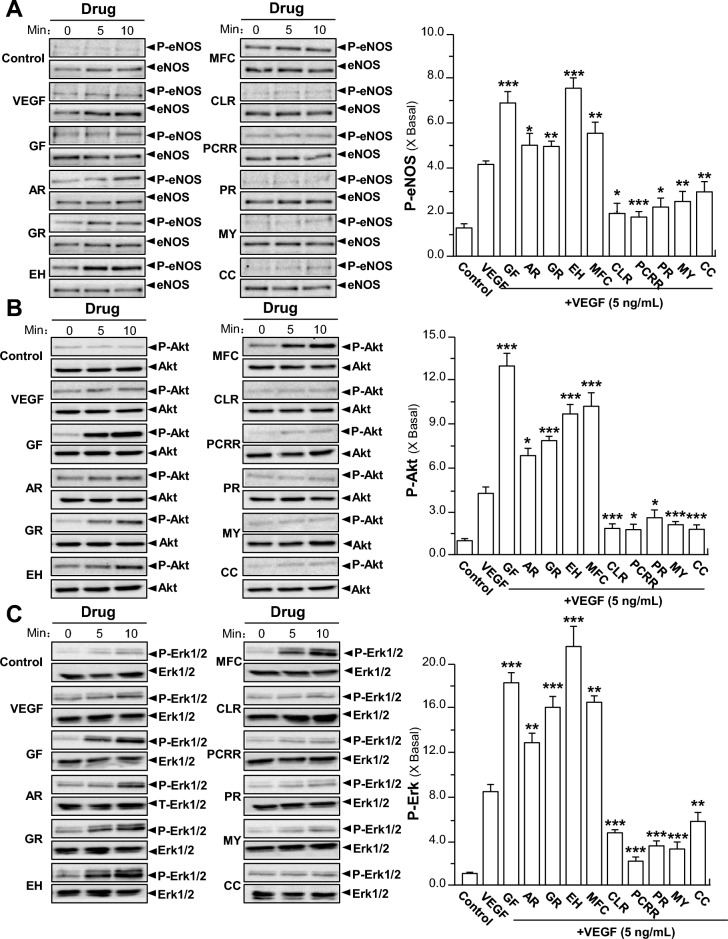
TCMs potentiate or suppress VEGF-induced phosphorylations of eNOS, Akt and Erk. A total of 20 × 10^4^ HUVECs per well were seeded in a 12-well plate. VEGF (5 ng/mL) was applied with or without GF, AR, GR, EH, MFC, CLR, PCRR, PR, MY, CC, as indicated. The cell lysates were collected after 10 min of treatment. Total and phosphorylated proteins of **A** eNOS, **B** Akt and **C** Erk were probed by western blotting assay (left panel). Abbreviations of herbs were described as in Fig. 5. Data are expressed as X Basal (right panel), where the control was set as 1, Mean ± SEM, where *n* = 3; *p* < 0.05 (*); *p* < 0.01 (**); *p* < 0.001 (***) vs VEGF-treated group

Compared with the control, the treatment of VEGF markedly increased the phosphorylation/activation of eNOS in a time-dependent manner: the maximal activation was at ~ 4-fold after 10 min. However, the separate application of Ginkgo Folium, Aucklandiae Radix, Glehniae Radix, Erigerontis Herba and Murrayae Folium et Cacumen effectively potentiated VEGF-induced phosphorylation of eNOS by ~ 2 to ~ 4-folds in a time-dependent manner, while the total expressions of them were not affected. Conversely, the separate treatment of Curcumae Longae Rhizoma, Polygoni Cuspidati Rhizoma et Radix, Polygalae Radix, Myrrha and Cinnamomi Cortex time-dependently inhibited VEGF-mediated eNOS phosphorylation by ~ 3-folds, with the total levels of them unaffected (Fig. 7A).

As shown in Fig. 7B, C, VEGF application, in HUVECs, induced the phosphorylations of Akt and Erk at ~ 5 and ~ 9-folds, separately. The total proteins of Akt and Erk were unchanged. Compared with VEGF-treated group, 5 herbs, i.e. Ginkgo Folium, Aucklandiae Radix, Glehniae Radix, Erigerontis Herba and Murrayae Folium et Cacumen, increased the phosphorylations of Akt and Erk separately by ~ 4 to ~ 15-folds. On the contrary, Curcumae Longae Rhizoma, Polygoni Cuspidati Rhizoma et Radix, Polygalae Radix, Myrrha and Cinnamomi Cortex significantly suppressed the VEGF-induced phosphorylations of Akt and Erk ~ 3 to ~ 7-folds. In addition, application of the 10 herbal extracts could evidently affect the VEGF-induced phosphorylations of Akt and Erk in time-dependent manners. The results of phosphorylations of Akt and Erk were similar to those obtained from eNOS phosphorylation investigations, further suggesting the effects of these TCM extracts on VEGF-mediated signaling.

### Summary of functional evaluations of TCMs

Effects of TCMs on VEGF-induced activities were tested in different functional assays regarding angiogenesis. The overall comparison among the ten herbs was summarized in Table 3. Among these herbs, Erigerontis Herba showed strongest activities in potentiating VEGF-mediated cell migration and Erk phosphorylation by ~ 60% and ~ 120%, respectively. Ginkgo Folium exhibited best potentiating effects on VEGF-induced tube formation and Akt phosphorylation with efficiency increased by ~ 80% and ~ 140%, respectively. For eNOS phosphorylation, both Ginkgo Folium and Erigerontis Herba enhanced VEGF-triggered activities by ~ 40%. In contrary, Higher activities in suppressing VEGF-mediated activities, including cell migration, tube formation, eNOS phosphorylation, Akt phosphorylation and Erk phosphorylation, were observed in Polygoni Cuspidati Rhizoma et Radix, with VEGF-induced efficiencies decreased by ~ 60% to ~ 80%, respectively. Curcumae Longae Rhizoma decreased VEGF-induced eNOS phosphorylation by ~ 80%. Based on these results, Ginkgo Folium and Polygoni Cuspidati Rhizoma et Radix, potentiating or suppressing VEGF responses, could be used to develop health products or drugs for angiogenesis-related diseases.

**Table 3:** The summary table of TCMs on wound healing, tube formation, eNOS phosphorylation, Akt phosphorylation and Erk phosphorylation

Name	Wound healing	Tube formation	eNOS phosphorylation	Akt phosphorylation	Erk phosphorylation
Ginkgo Folium	↑↑	↑↑↑↑	↑↑	↑↑↑↑↑↑↑	↑↑↑↑↑
Aucklandiae Radix	↑↑	↑↑	↑	↑↑	↑↑
Glehniae Radix	↑	↑↑	↑	↑↑↑	↑↑↑
Erigerontis Herba	↑↑↑	↑↑↑	↑↑	↑↑↑↑	↑↑↑↑↑↑
Murrayae Folium et Cacumen	↑	↑↑	↑	↑↑↑↑	↑↑↑
Curcumae Longae Rhizoma	↓↓	↓↓	↓↓↓↓	↓↓↓	↓↓
Polygoni Cuspidati Rhizoma et Radix	↓↓↓	↓↓↓	↓↓↓↓	↓↓↓	↓↓↓↓
Polygalae Radix	↓	↓↓	↓↓↓	↓↓	↓↓↓
Myrrha	↓	↓	↓↓	↓↓↓	↓↓↓
Cinnamomi Cortex	↓	↓↓	↓	↓↓↓	↓

One piece of “↑” represents certain kind of herb extract could increase VEGF-mediated functions by ~ 20%; while one piece of “↓” stands for certain kind of herb extract could inhibit VEGF-mediated functions by ~ 20%; for example, “↑↑↑↑” showed in the second row represents Ginkgo Folium could promote VEGF-induced tube formation by ~ 80%

## Discussion

HerboChips is a new platform for reverse screening of the effects of TCMs, a U.S. patent filed and applied for by Chang et al. in 2003 and later improved by Huang et al. in 2015; the principle of the platform is based on microarrays where known protein drugs are targeted and conjugated to a chip containing multiple compounds, which are then hybridized using Cy5-labelled streptavidin (SA-Cy5) and finally fluorescence intensity is observed at 635 nm [24, 31, 37]. Currently, the HerboChips platform has elucidated the function of many TCMs on known disease targets, such as the inhibitory function of *Geranium wilfordii* on NGF and TNF-α, and the inhibitory function of green tea on α-glucosidase [24, 34, 35, 45]. In this study, we demonstrate for the first time the applicability of the HerboChips platform for VEGF targets. In order to obtain hundreds of different herbal extracts in a short time, though there exist some limitations, 50% ethanol was uniformly applied in the extraction process as ethanol is a common organic solvent used in extraction of active components from TCMs [46, 47]. Based on the screening results of more than 500 microarrays, we identified 38 TCMS extracts presenting positive hits with VEGF. Then, based on toxicity and accessibility, we further selected Ginkgo Folium, Aucklandiae Radix, Glehniae Radix, Erigerontis Herba, Murrayae Folium et Cacumen, Curcumae Longae Rhizoma. Polygoni Cuspidati Rhizoma et Radix, Polygalae Radix, Myrrha and Cinnamomi Cortex, which are the 10 TCMs to further elucidate the effect on VEGF and to validate the screening results. Notably, these TCMS have been traditionally used based on specific functions such as angiogenesis, anti-inflammatory and anti-cancer properties, e.g. *Ginkgo biloba* suppresses cancer cell invasion and angiogenesis through attenuation of angiogenin [48]; Scutellarin, the main active compound of *Erigerontis Herba*, represents a potential candidate for cancer treatment via its angiogenic functions [49]. Polygoni Cuspidati Rhizoma et Radix can inhibit VEGF-induced angiogenesis [24]; *Curcuma longa* extract can suppress angiogenesis based on a mouse model of wound healing [50]. However, the molecular mechanisms of VEGF-induced angiogenesis by these 10 TCMs have not been precisely reported.

It is well known that angiogenesis is a complex process that requires growth factors, cytokines, and many other factors to coordinate and act on each other [51]. Although there are numerous factors influencing this process, it is VEGF-mediated endothelial cell proliferation, migration, invasion and survival that play a major role [52]. It has been demonstrated that VEGF regulates endothelial cell proliferation, migration, invasion and survival mainly through binding to its receptor (VEGFR) [53, 54]. When VEGF binds to VEGFR, it leads to a conformational change in VEGF/VEGFR and triggers dimerisation and autophosphorylation of complex amino acid residues, which activates downstream signalling pathways such as PI3K/Akt, MAPK/Erk and eNOS [4, 5, 55, 56]. The functions of signalling pathways, e.g. Akt, Erk and eNOS, in endothelial cells have been well elucidated [57, 58], with Akt being responsible for endothelial cell survival, Erk for endothelial cell proliferation and eNOS for endothelial cell migration and vascular permeability. In addition, Akt can affect the phosphorylation level of eNOS and the release of NO [59, 60], which in turn affects the expression of VEGF [61]. Given the importance of Akt, Erk and eNOS for VEGF-mediated angiogenesis, we hypothesised that these 10 herbal medicines affect VEGF-mediated cell proliferation, migration, invasion and angiogenesis through downstream signalling pathways such as Akt, Erk and eNOS.

To test this hypothesis, we conducted a series of studies with cultured HUVEC cells. The results, as expected, were that the 10 TCMs exerted their promotional or inhibitory effects on VEGF-mediated cell proliferation, migration, invasion, and angiogenesis via Akt, Erk, and eNOS, which are consistent with previous studies. At present, among the approved anti-VEGF drugs, such as sunitinib and ranibizumab, they act through mechanisms that block or attenuate VEGF/VEGFR binding activity or downstream signalling pathways such as Akt, Erk and eNOS [53, 62]. But, these TCM extracts do not act on the target as accurately as these targeted synthetic drugs that inhibit VEGF, because these TCM extracts contain a very large number of natural compounds, some of which, such as phenols and terpenes, have great potential for anti-angiogenesis, whereas others do not [63]. The inhibitory effects of five kinds of herbal extracts in VEGF-treated cultures may be attributed to their occupation of possible binding sites between VEGF and VEGFR, which subsequently reduced the amount of VEGF binding to VEGFR. However, the left herbal extracts possessing potentiating VEGF-mediated angiogenic functions, enhanced the VEGF-triggered phosphorylations of VEGFR2 and downstream molecules. The potentiating activities of these herbal extracts might be accounted by their direct bindings with VEGF at the heparin binding domain, i.e. to promoting the binding of VEGF to its receptor, which could be different from binding sites of the interactions of VEGF-five herbal extracts (Curcumae Longae Rhizoma, Polygoni Cuspidati Rhizoma et Radix, Polygalae Radix, Myrrha and Cinnamomi Cortex). Thus, the herbal extracts with potentiating effects on VEGF-induced angiogenesis could be used for treatment of ischemic heart disease related with angiogenesis, including coronary heart disease and myocardial infarction.

But, these targeted VEGF-inhibiting drugs are synthetic drugs, whereas TCM contains many kinds of natural compounds, such as flavonoids and alkaloids [63]. In addition, studies have shown that active ingredients in TCMs, such as phenols and terpenoids, have great potential in anti-angiogenesis [63]. Unfortunately, we have not been able to identify the active ingredients of these herbs that have an effect on VEGF by optimizing the extraction method for each kind of TCM to obtain high extraction rate or trying different chromatographic methods to improve related resolution. Though we have recorded certain kinds of compounds existing in the herbal extracts to confirm the repeatability of all biochemical studies, content determination for each compound hasn’t been conducted. But in the future, we will refine this aspect of our programme to focus on the active ingredients of these TCMs.

In recent years, with the clinical use of anti-VEGF-targeted drugs, a number of very serious drawbacks have been highlighted, such as side effects like thrombosis and hypertension, expensive treatment and drug resistance [12, 63]. As a result, the screening of potential drugs against VEGF now favors the selection of natural drugs or natural compounds that are effective, have few side effects and are affordable [17, 63, 64]. Based on these findings, our study demonstrated for the first time the applicability of the HerboChips platform to VEGF targets and clarified the roles of these 10 TCMs in VEGF-mediated angiogenesis and the related signalling mechanisms. Meanwhile, the clinical application of the above anti-VEGF drugs also provides further support and assistance for the development of potential drugs of these 10 TCMs in the treatment of angiogenesis or related diseases. In addition, during wound healing, VEGF helps various substances to pass through the blood vessels, which in turn forms barriers and neovessels to stop the invasion of pathogens and aid in wound healing [43, 65]. Therefore, we believe that herbal medicines with a promoting effect on VEGF can be used to develop drugs that promote wound healing.

## Conclusions

Our results, for the first time, demonstrated the applicability of the HerboChip platform for screening potential drugs on VEGF targets and further elucidated the activity of the 10 Chinese medicines on angiogenesis in vitro and the related signal transduction mechanisms. These results, meanwhile, provide further support and assistance for the development of these 10 Chinese medicines in the treatment of angiogenesis or related diseases and pro-wound healing drugs.

## Supplementary Information

Supplementary Figure S1. Binding signals of representative seven kinds of HerboChips probed by VEGF. The images were visualized by Cy5-labeled streptavidin after the binding of VEGF to AR-, GR-, EH-, CLR-, PCRR-, PR- and CC-HerboChips, which were fabricated with different kinds of extracts, respectively. Abbreviations of herbs were described as in Fig. 5. Supplementary Figure 2. TCMs exert effects on VEGF-induced cell proliferation. Different concentrations of herbal extracts were applied onto endothelial cells for 48 h, and MTT assay were determined. Data are demonstrated as Mean ± SEM of the percentage of change as compared to control group, where *n* = 4; *p* < 0.05; *p* < 0.01; *p* < 0.001vs control group. Abbreviations of herbs were described as in Fig. 5. Supplementary Figure 3. Representative pictures showing the effects of seven kinds of herbs on VEGF-induced cell migration. Abbreviations of herbs were described as in Fig. 5. Supplementary Figure 4. Representative pictures showing the effects of seven kinds of herbs on VEGF-induced tube formation. Abbreviations of herbs were described as in Fig. 5.

## Data Availability

All datasets generated for this study are included in the article/Supplementary Material.

## Abbreviations

VEGF: Vascular endothelial growth factor
TCM: Traditional Chinese medicine
HUVECs: Human umbilical vein endothelial cells
VEGFR: VEGF receptor
PI3K: Phosphoinositide 3-kinase
Akt: Protein kinase B
MAPK: Mitogen-activated protein kinase
Erk: Extracellular signal-regulated kinase
eNOS: Endothelial-type nitric oxide synthase
NGF: Nerve growth factor
TNF-α: Tumor necrosis factor α
CYP450 3A4: Cytochrome P450 3A4
NF-κB: Nuclear factor kappa-light-chain-enhancer of activated B cells
STAT3: Signal transducer and activator of transcription 3
GF: Ginkgo Folium
AR: Aucklandiae Radix
GR: Glehniae Radix
EH: Erigerontis Herba
MFC: Murrayae Folium et Cacumen
CLR: Curcumae Longae Rhizoma
PCRR: Polygoni Cuspidati Rhizoma et Radix
PR: Polygalae Radix
MY: Myrrha
CC: Cinnamomi Cortex
