# Supplementary material for: Screening of herbal extracts binding with vascular endothelial growth factor by applying HerboChip platform
Source: Chin Med. 2024 Sep 9;19:122. doi: 10.1186/s13020-024-00987-x (PMC11382504; doi:10.1186/s13020-024-00987-x)
Supplement: Supplementary file 1 — Supplementary Figure S1. Binding signals of representative seven kinds of HerboChips probed by VEGF. The images were visualized by Cy5-labeled streptavidin after the binding of VEGF to AR-, GR-, EH-, CLR-, PCRR-, PR- and CC-HerboChips, which were fabricated with different kinds of extracts, respectively. Abbreviations of herbs were described as in Fig. 5. Supplementary Figure 2. TCMs exert effects on VEGF-induced cell proliferation. Different concentrations of herbal extracts were applied onto endothelial cells for 48 h, and MTT assay were determined. Data are demonstrated as Mean ± SEM of the percentage of change as compared to control group, where n = 4; p < 0.05; p < 0.01; p < 0.001vs control group. Abbreviations of herbs were described as in Fig. 5. Supplementary Figure 3. Representative pictures showing the effects of seven kinds of herbs on VEGF-induced cell migration. Abbreviations of herbs were described as in Fig. 5. Supplementary Figure 4. Representative pictures showing the effects of seven kinds of herbs on VEGF-induced tube formation. Abbreviations of herbs were described as in Fig. 5. [file 13020_2024_987_MOESM1_ESM.pdf]

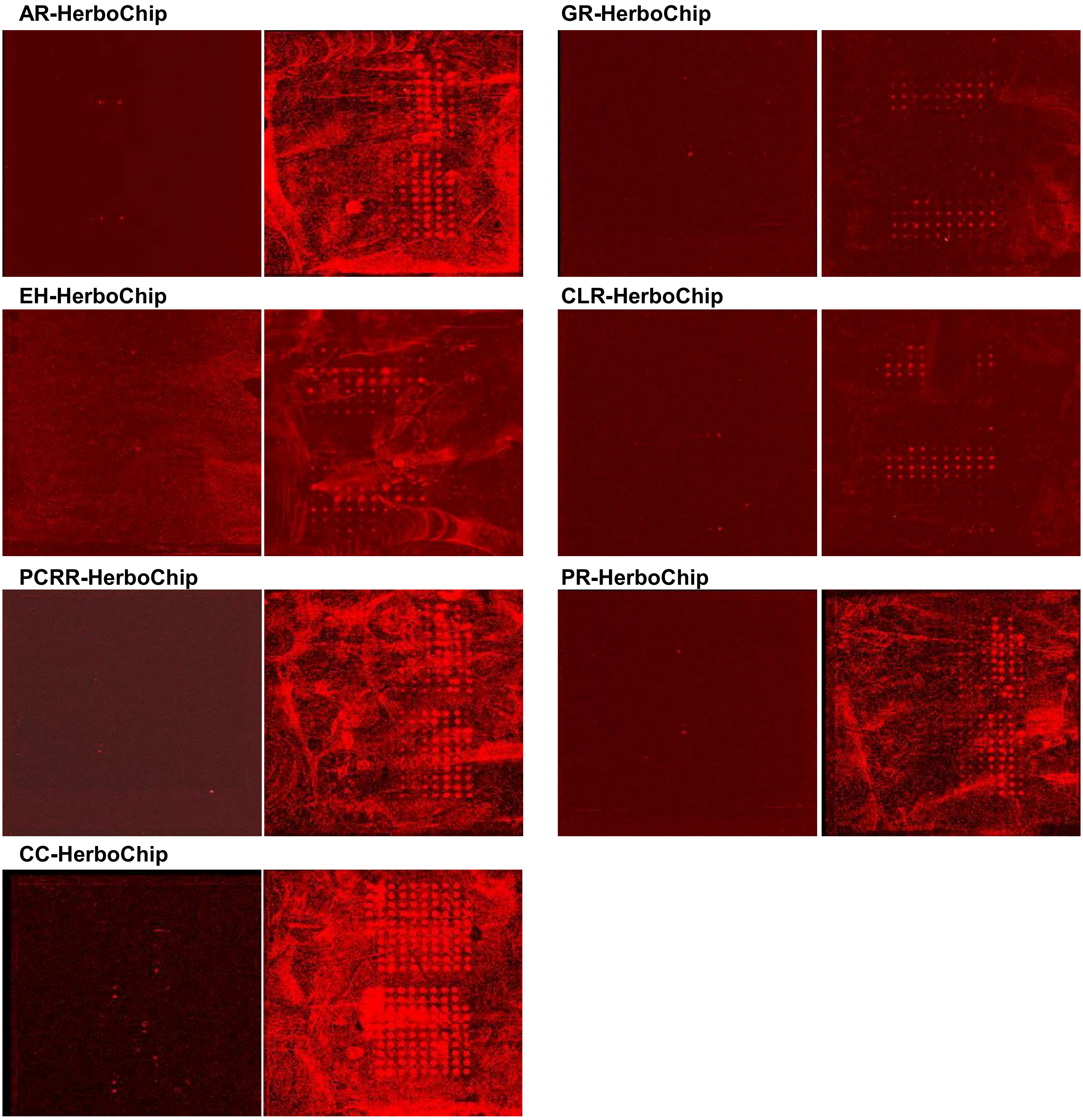

Supplementary Fig. 1

Liu et al 2024

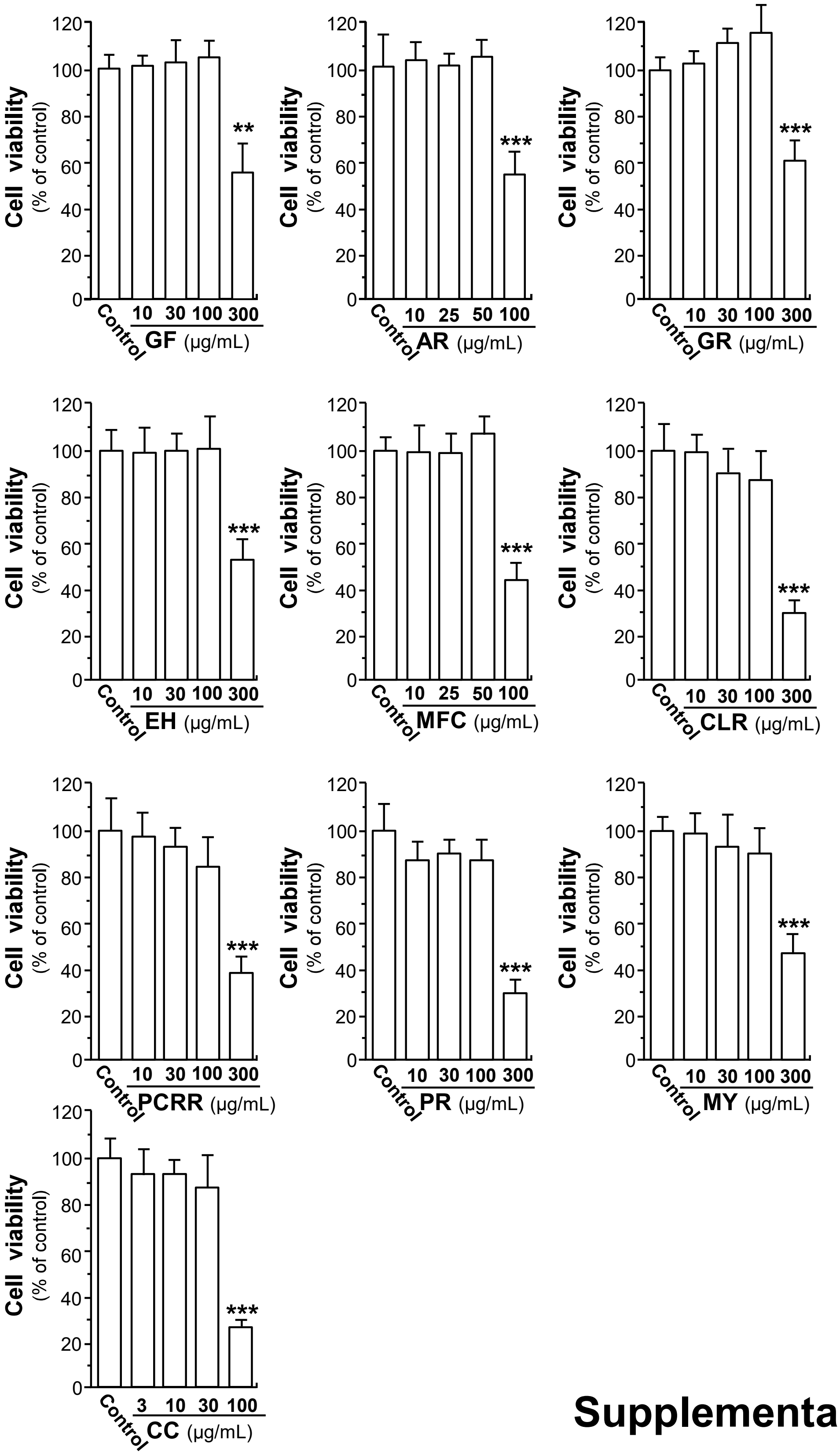

Supplementary Fig. 2

Liu et al 2024

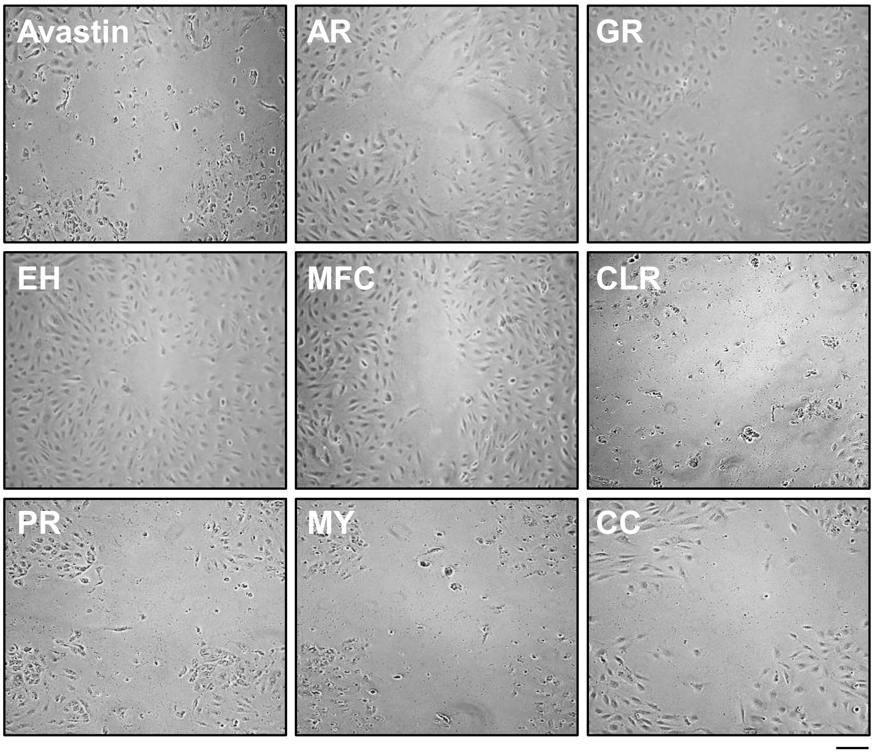

Supplementary Fig. 3
Liu et al 2024

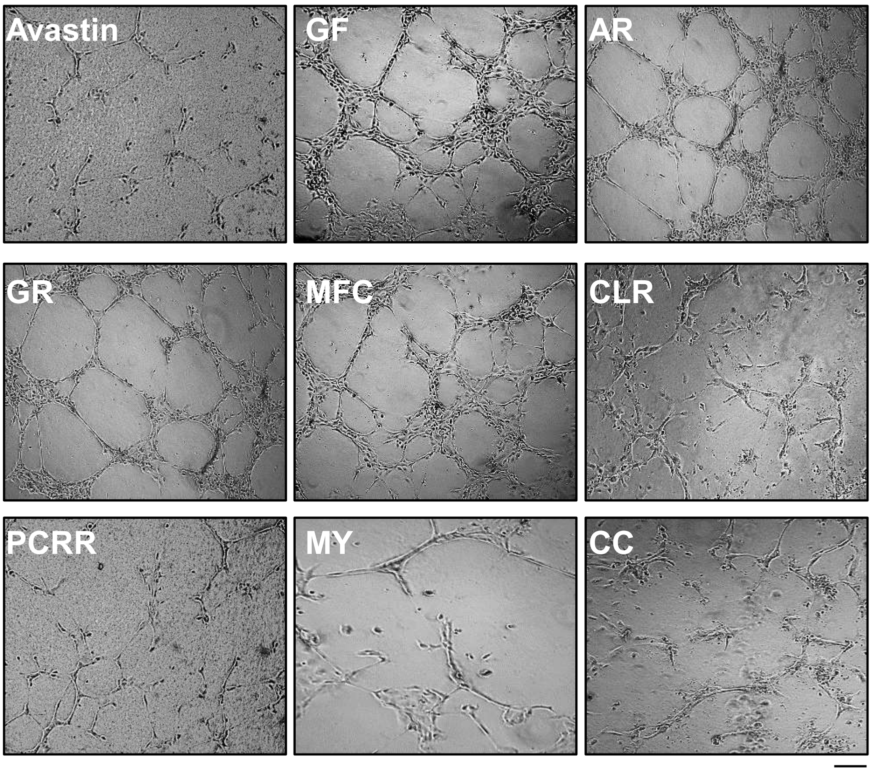

Supplementary Fig. 4

Liu et al 2024
